# Trajectories and predictors of the long-term course of low back pain: cohort study with 5-year follow-up

**DOI:** 10.1097/j.pain.0000000000001097

**Published:** 2017-11-03

**Authors:** Ying Chen, Paul Campbell, Victoria Y. Strauss, Nadine E. Foster, Kelvin P. Jordan, Kate M. Dunn

**Affiliations:** a Arthritis Research UK Primary Care Centre, Research Institute for Primary Care and Health Sciences, Keele University, Staffordshire, United Kingdom; b Nuffield Department of Orthopaedics, Rheumatology and Musculoskeletal Sciences, Centre for Statistics in Medicine, Botnar Research Centre, University of Oxford, Oxford, United Kingdom; c Keele Clinical Trials Unit, Keele University, Staffordshire, United Kingdom

**Keywords:** Low back pain, Latent class analysis, Pain trajectory, Prognostic factor

## Abstract

Low back pain (LBP) is a major health challenge globally. Research has identified common trajectories of pain over time. We aimed to investigate whether trajectories described in 1 primary care cohort can be confirmed in another, and to determine the prognostic value of factors collected 5 years prior to the identification of the trajectory. The study was conducted on 281 patients who had consulted primary care for LBP, at that point completed a baseline questionnaire, and then returned a questionnaire at 5-year follow-up plus at least 3 (of 6) subsequent monthly questionnaires. Baseline factors were measured using validated tools. Pain intensity scores from the 5-year follow-up and monthly questionnaires were used to assign participants into 4 previously derived pain trajectories (no or occasional mild, persistent mild, fluctuating, and persistent severe), using latent class analysis. Posterior probabilities of belonging to each cluster were estimated for each participant. The posterior probabilities for the assigned clusters were very high (>0.90) for each cluster except for the smallest “fluctuating” cluster (0.74). Lower social class and higher pain intensity were significantly associated with a more severe trajectory 5 years later, as were patients' perceptions of the greater consequences and longer duration of pain, and greater passive behavioural coping. Low back pain trajectories identified previously appear generalizable. These allow better understanding of the long-term course of LBP, and effective management tailored to individual trajectories needs to be identified.

## Abstract 2

Supplemental Digital Content is Available in the Text.

Low back pain trajectories identified previously appear generalizable. Effective management tailored to individual trajectories needs to be identified.

## 1. Introduction

Low back pain (LBP) is common. It is the leading cause of years lived with disability worldwide.^39^ It also has a major impact on health services because 25% to 30% of people with back pain will consult their general practitioner about their pain each year.^35^ Most consulters will not seek health care beyond the first 3 months, although up to 80% still have pain or disability a year later.^8,22^ Many people with back pain experience pain over a number of years,^13,22^ but despite this, few studies include follow-up beyond a 1-year period.^3,6,18^

In our previous work among primary care patients with back pain, we identified, for the first time, 4 trajectories of change in back pain over time: persistent mild, recovering, severe, and fluctuating.^15^ In the long-term follow-up of that cohort, we have shown evidence that these trajectories persist over many years.^11^ Other studies have since also described trajectories of back pain.^25^ Despite some differences between studies, common trajectories have been identified across settings and countries. However, no research investigated if the patterns already described in 1 cohort can be confirmed in new cohorts.^25^ We had the opportunity to replicate methods we have previously used in 1 cohort (BaRNS study),^11,15^ within the follow-up of a separate cohort of primary care patients with back pain (BeBack study),^19^ thereby facilitating examination of the generalisability of findings between samples, and allowing investigation of the potential for wider use and application of the findings.

Predictors of back pain outcome have been identified in a range of studies, but these studies have commonly used the presence or level of back pain at a single point as the outcome.^30,40^ Studies have described associations with identified trajectories,^2,7,9–11,15,26,29,37^ but none, to date, have been able to determine predictors of trajectory membership at a time point prior to the identification of the trajectory. This is important to establish a clear time sequence between the predictive factor and the outcome (in this case, a trajectory).

The aims of this study were to, therefore, investigate whether back pain trajectories found in 1 cohort of patients with LBP consulting in primary care are observed in a separate sample, and whether predictors of those trajectories can be identified.

## 2. Methods

### 2.1. Study design and setting

This was a prospective cohort study of patients seeking health care for LBP in 8 general practices within the North Staffordshire and Cheshire area, England (BeBack Study). Consecutive adults aged 18 to 60 years, who visited their general practitioner about back pain between September 2004 and April 2006, were sent information about the study and invited to take part. Further details about recruitment are reported elsewhere.^19^ Ethical approval for all phases of the study was obtained from the North Staffordshire and North West Cheshire Research Ethics Committees.

A total of 1591 participants participated in the cohort at the initial baseline.^19^ The eligible subjects for this 5-year follow-up study were derived from 1289 patients who responded to the initial baseline questionnaire and gave permission for further contact; 810 (63%) responded again after 6 months, and 696 of these (86%) were traced and contacted 5 years later. This eligible sample was sent a questionnaire at the 5-year follow-up stage, followed by 6 shorter monthly questionnaires. In total, 488 responded at the 5-year follow-up stage (70%) and 281 (40%) completed the 5-year follow-up questionnaire and at least 3 subsequent monthly questionnaires. Participants in this analysis were those 281 patients.

### 2.2. Data collection

In all questionnaires, back pain intensity was derived from the mean of 3 self-reported 11-point numeric rating scales (0-10) for the least and usual pain in last 2 weeks, and current pain.^16^ Physical disability associated with back pain was measured using the Roland–Morris Disability Questionnaire (24 items, score range 0-24).^36^ Pain duration was measured as time since the last pain-free month,^13^ and the presence of leg pain and distal leg pain was reported for the previous 2 weeks. These are classified as pain-related factors.

Psychological factors were selected based on previous prognostic findings within the 2004 to 2006 data set.^4,5,19,20^ These were measured in the initial baseline questionnaire, using the Illness Perception Questionnaire-Revised.^31^ The Illness Perception Questionnaire-Revised contains 5 subscales relating to the illness (in this case pain): consequences (the consequences related to pain, score range 6-30), emotional representation (the emotional impact of pain, score range 6-30), personal control (how much perceived control the person has on the management of their pain, score range 6-30), treatment control (how much perceived control for the pain can be attributed to treatments, score range 5-25), and timeline (beliefs on how long the condition will last, score range 6-30). The Coping Strategies Questionnaire 24 was used to assess the level of catastrophising in relation to pain (catastrophizing subscale, 6 items, score range 0-36),^21^ the Hospital Anxiety and Depression Scale was used to measure affect (14 questions, score range 0-21 for anxiety and depression separately),^42^ the Tampa Scale for Kinesiophobia was used to measure fear of movement (TSK, 17 items, score range 17-68),^27^ and the Pain Self-efficacy Questionnaire was used to assess the ability of the person to cope and manage despite their current pain levels (10 items, score range 0-60).^33^ Finally, passive behavioural coping items were included measuring aspects such as withdrawal from activities, avoidance, and resting (6 items, score range 0-6).^41^

Baseline questionnaires also included the sociodemographic and occupational factors of age, sex, educational level (education up to the age of 16 years vs education beyond age 16), social class (higher: managerial, professional, intermediate, self-employed occupations vs lower: supervisory, technical, semi-routine, and routine occupations), and current working status (working as normal vs reduced work or not working).

### 2.3. Statistical analysis

From the 5-year questionnaire and the subsequent 6 monthly questionnaires, pain intensity scores were trichotomized into no pain (a score <1.0), mild-moderate pain (a score ≥1.0 and <5.0), and high pain (a score ≥5.0), analysed as an ordinal variable. This cutoff has been established in our previous studies^15,19^ and is supported by evidence that individuals scoring less than the midpoint on a pain intensity scale were unlikely to suffer a significant level of disability.^38^ Questionnaires were scored according to the systems suggested by the developers, where appropriate.

Baseline characteristics were grouped by domain: sociodemographic and occupational (age, sex, education, social class, and employment status), pain-related (pain intensity, disability, pain duration, leg pain, and distal pain), and psychological (illness perceptions, depressive and anxiety symptoms, fear of movement, catastrophising, coping, and self-efficacy), similar to previous analyses.^5^

#### 2.3.1. Assignment of individuals to trajectories

The categorised pain intensity scores from the 5-year questionnaire and the following 6 monthly questionnaires were used to cluster participants into different courses of pain, using longitudinal latent class analysis (LLCA), as in the BaRNS study.^15^ The assumption behind latent class analysis is that there exist a certain number of distinct pathways of LBP, and participants can be grouped into a small number of clusters representing these pathways based on their profiles of pain over time, with each participant belonging to 1 cluster. The 4 trajectories (“no or occasional mild,” “persistent mild,” “persistent severe,” and “fluctuating between mild and severe pain”) identified at 7-year follow-up from 112 participants in the BaRNS study^11^ were used as the basis for this analysis, and each of the BeBack study participants were allocated to the predefined cluster best matching their pain profile. To do this, the 281 BeBack participants were merged into a single data set with the 112 participants from the BaRNS study, who were preclassified into their LLCA clusters. A 4-class restricted LLCA model was applied based on the 4 pre-established clusters. The posterior probabilities of belonging to each of the 4 clusters for the BeBack participants were then freely estimated within this model. Participants were allocated to the cluster for which they had the highest probability. The goodness of fit of the model was assessed by determining the mean posterior probabilities for the BeBack study participants allocated to each cluster, and subjective assessment of how well individual trajectories within a cluster followed the cluster-specific trajectory. Participants should be allocated to their assigned cluster with a high probability of belonging to that cluster; lower probabilities might suggest that the model has difficulty discriminating between clusters and that participants may not match the trajectory described by their assigned cluster. Mean posterior probabilities above 0.70 are generally considered to show clear allocation of participants to clusters.^32^ LatentGOLD 4.0 was used for this analysis.

An alternative approach to assess the generalizability of the previously derived trajectories is to assess whether we would identify the same number of clusters and trajectory patterns for this cohort using the same modelling method used in the previous study.^11,15^ However, there is no definitive method of identifying the best fitting model, and so, both statistical goodness of fit indices (of which there are several) and interpretation of the resultant clusters are generally used. This means selection of the optimal model is somewhat subjective with potential for bias through our knowledge of the trajectories identified in our previous study. Therefore, we carried this out purely as a sensitivity analysis by first using statistical goodness of fit indices to assess whether a 4-cluster model appeared optimal for this cohort. We then used the monthly cluster-specific probabilities of having each level of pain to assess whether these 4 clusters yielded similar trajectories as in the previous study. See supplementary file 1 for full details of the methods (available online at http://links.lww.com/PAIN/A499).

#### 2.3.2. Determination of prognostic factors

To determine factors predictive of pain course at 5-year follow-up, we used a stepped process based on an approach we have used previously.^23,24^ Possible collinearity between potential prognostic factors was tested. Unadjusted relative risk ratios (RRRs) were calculated (with 95% confidence intervals [CIs]) to show the univariable association between each potential prognostic factor and the 5-year cluster group using univariable multinomial logistic regression models. Multivariable multinomial logistic regression modelling was then used within each domain (sociodemographic and occupational, pain-related, and psychological) to assess the independent associations of the significant factors (statistical significance of any level of the ordinal variable) from the univariable analysis with pain course at 5 years. Then, all significant variables in the within-domain analyses were included in a final model, with all variables entered simultaneously. The “no or occasional mild” group was set as the reference group. Given the small prevalence of the fluctuating trajectory in the BaRNS study^11^ and the relative small cohort size of this study, the fluctuating group was combined with the “persistent severe” group.

We further determined whether the baseline prognostic factors had similar relationships with a single assessment (ie, pain intensity score at 5 years) as identified for the patient clusters based on multiple assessments (ie, the pain trajectories). Additional multinomial logistic regression models using the same stepped approach but using the trichotomized pain score at 5 years (<1.0 as no pain, ≥1.0 and <5.0 as mild-moderate pain, and ≥5.0 as high pain) as the dependent variable were performed. Analysis was performed using STATA 14 (StataCorp LLC, TX).

## 3. Results

Characteristics of the included sample from their initial baseline BeBack study questionnaires are presented in Table 1. Comparing these participants with patients who responded to the 5-year questionnaire but did not return enough subsequent monthly questionnaires (n = 207) showed only significant difference on age. Participants in this analysis were slightly (mean 48 vs 46) older (see supplementary file 2, available online at http://links.lww.com/PAIN/A499).

**Table 1:**
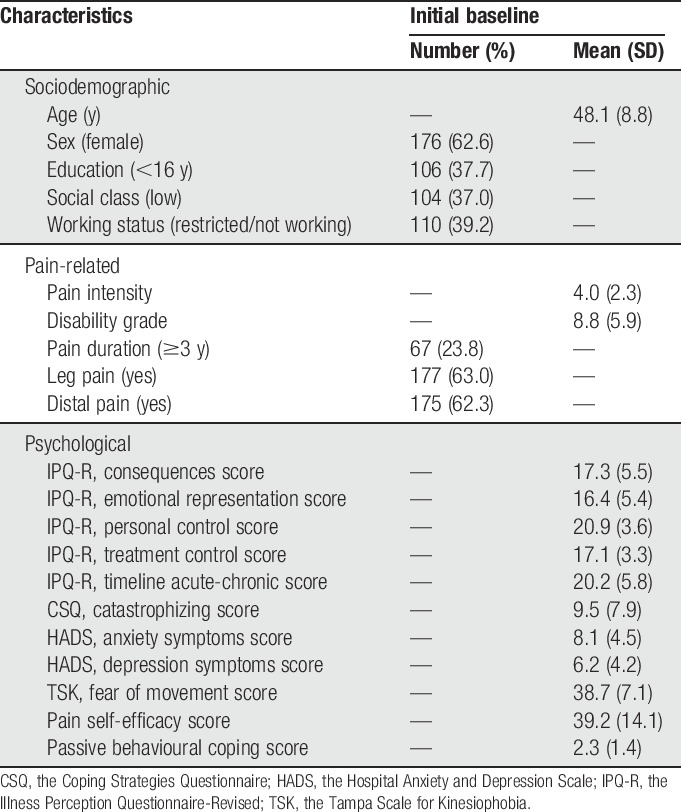
Characteristics of participants at initial baseline (n = 281).

### 3.1. Trajectories analysis

The 281 participants in the current analysis were allocated to the 4 predefined clusters using LLCA. Seventy nine (28%) were included in the “no or occasional mild” pain cluster, 131 (47%) in the “persistent mild” cluster, 60 (21%) in the “persistent severe” cluster, and 11 (4%) in the “fluctuating” cluster. The mean posterior probabilities for the assigned clusters were over 0.90 for each cluster except for the fluctuating cluster where it was 0.74. The probability of belonging to each nonassigned class was under 0.10 except for those allocated to the fluctuating cluster that had a mean probability of 0.22 of being allocated to the persistent mild cluster (Table 2). This suggests that the clusters were distinct and participants were clearly allocated to their assigned cluster.

**Table 2:**
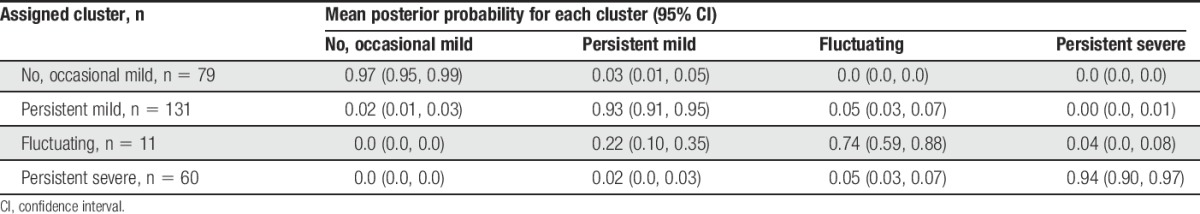
Posterior probability of membership of clusters (n = 281).

**Figure 1.**
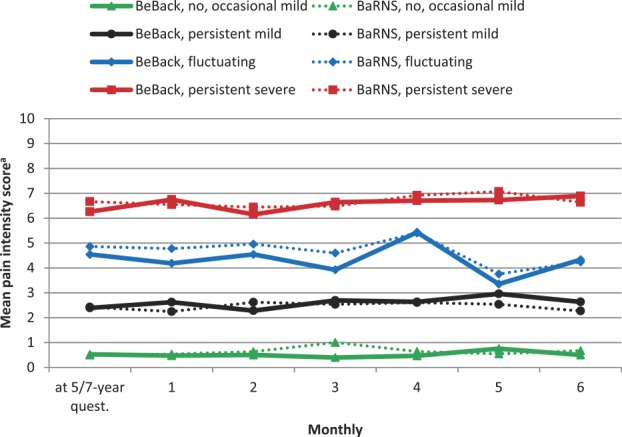
Mean monthly back pain intensity scores of current study participants (BeBack, 5-year follow-up) and the comparison study (BaRNS, 7-year follow-up). ^a^Original score on an 11-point scale (0-10).

The mean monthly pain intensity scores (trajectories) for each of the clusters have been plotted in Figure 1, and the clearly separate trajectories for the different clusters are apparent. Trajectories for the current analysis (BeBack study participants with 5-year follow-up) as well as the previous analysis (BaRNS study participants with 7-year follow-up)^11^ are shown and indicate very similar monthly cluster-specific mean scores in the 2 cohorts.

Comparison of the initial baseline characteristics of participants in the clusters at 5-year follow-up indicates that people in milder clusters were more highly educated and less likely to not work or have reduced their work than those in more severe clusters. Participants allocated to the milder clusters also reported shorter pain duration, less leg pain, and had lower scores on all of the measures of psychological factors (Table 3).

**Table 3:**
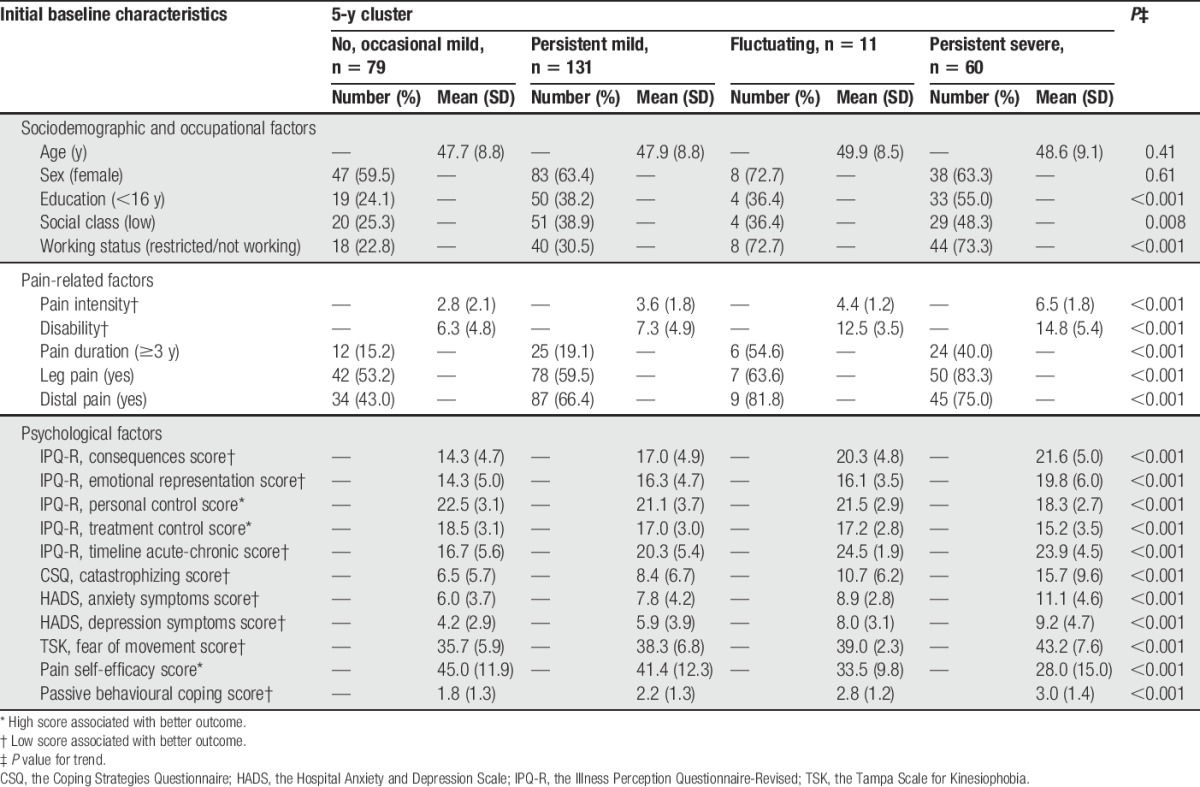
Initial baseline characteristics of 281 patients with low back pain stratified by trajectory clusters at 5 years.

### 3.2. Prognostic factors

All the selected baseline factors, except for age and sex, were found to be associated with 5-year cluster group in the univariable analyses (Table 4). After adjustment within each domain, social class and working status (from sociodemographic and occupational domain), pain intensity, physical disability, pain duration and distal pain (from pain-related domain), perceived consequence, emotional representation, personal control, patient's perception that the pain will last a long time, anxiety, and passive behavioural coping (from psychological domain) were still associated (Table 4).

**Table 4:**
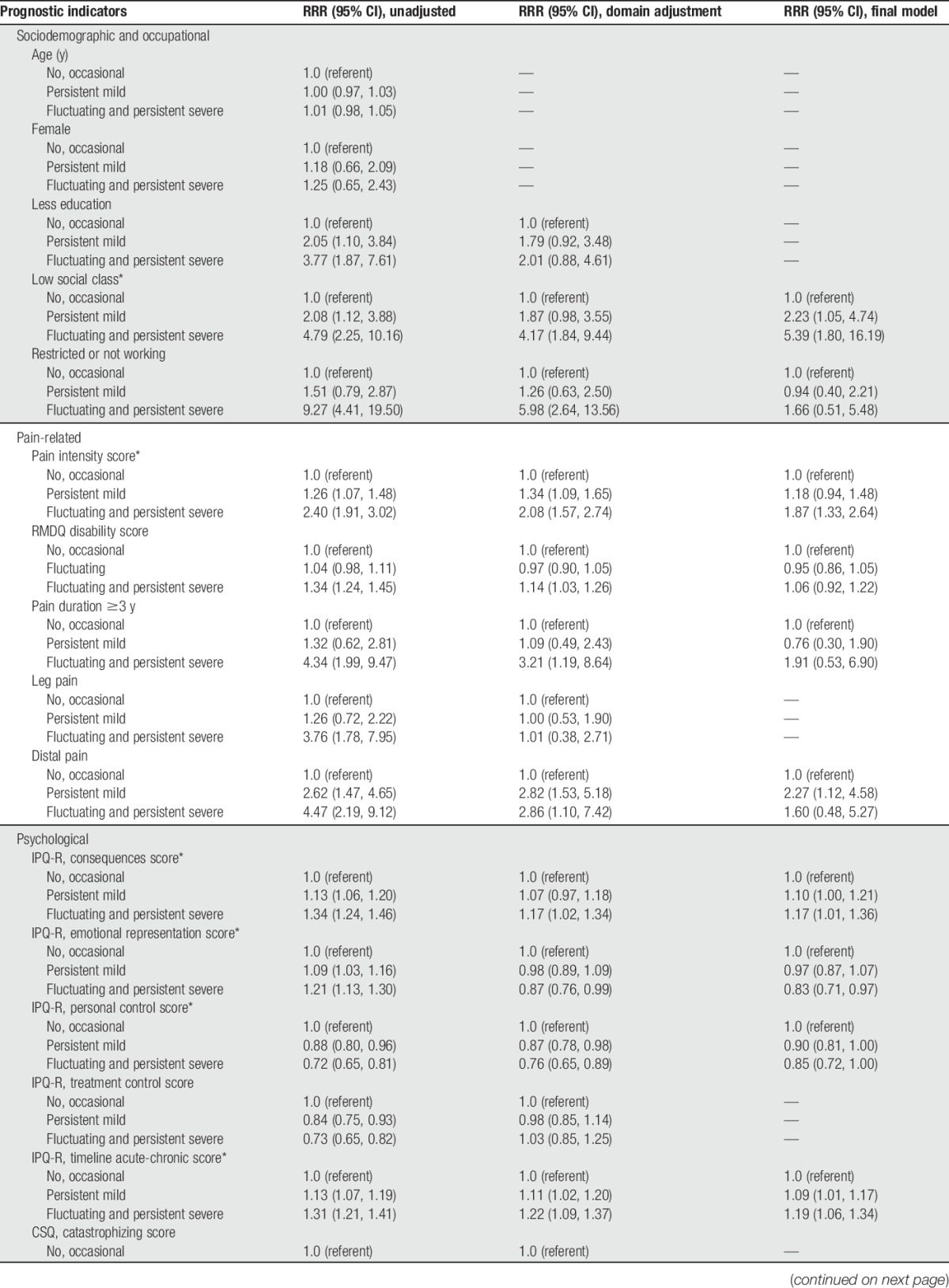

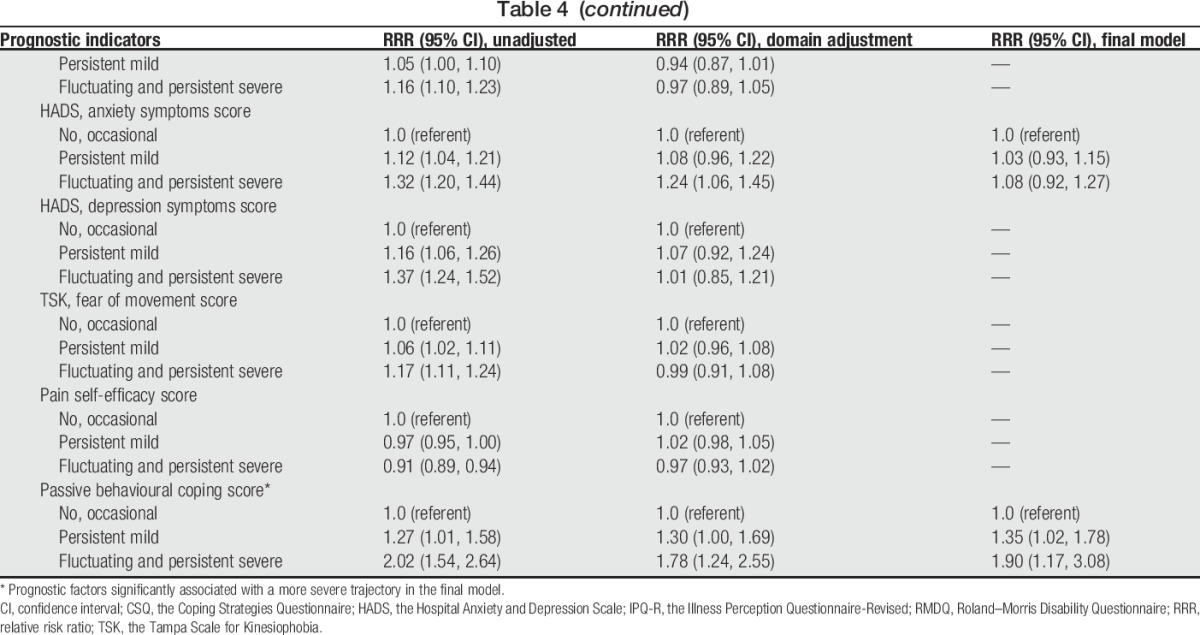
Multinomial logistic regression models for the relationship between potential prognostic indicators at initial baseline and membership of pain trajectories clusters at 5 years.

In the final model, the baseline factors significantly associated with more severe 5-year pain course were as follows: lower social class (RRR 5.4, 95% CI 1.8-16.2; “persistent severe” and “fluctuating” to “no, occasional”), higher pain intensity (RRR 1.9 per unit increase, 95% CI 1.3-2.6), greater perception on serious consequence from pain (RRR 1.2 per unit increase, 95% CI 1.0-1.4), lower emotional representation (RRR 0.8 per unit increase, 95% CI 0.7-1.0), greater perception that the pain will last a long time (RRR 1.2 per unit increase, 95% CI 1.1-1.3), less beliefs in the personal controllability of pain (RRR 0.9 per unit increase, 95% CI 0.7-1.0), and a higher passive behavioural coping score (RRR 1.9 per unit increase, 95% CI 1.2-3.1) (Table 4).

Statistically significant predictors of a worse 5-year outcome when based on a single assessment (ie, pain intensity score at 5 year) were higher baseline pain intensity, longer pain duration, greater perception that the pain will last a long time, and a higher passive behavioural coping score (supplementary file 3, available online at http://links.lww.com/PAIN/A499).

### 3.3. Sensitivity analysis

The sensitivity analysis deriving latent classes for this cohort using the same approach as in the original study showed that a 4-cluster model fitted this cohort's data well. The derived clusters were similar in their patterns of pain as the original clusters. The mean posterior probabilities for the assigned clusters were over 0.95 for each cluster except for the “fluctuating” cluster, where it was 0.88. The probability of belonging to each nonassigned cluster was low (<0.12). Comparison of the assignment of participants to the clusters to their cluster assignments based on the previously identified clusters used in the main analysis showed that 259/281 (92%) participants were assigned to the same clusters (see supplementary file 1, available online at http://links.lww.com/PAIN/A499).

## 4. Discussion

This study shows that LBP trajectories identified within 1 primary care consultation cohort are generalizable to another. Predictors of those trajectories, apparent 5 years before the identification of the trajectories, have also been identified. It is the first time that the external validity of identified trajectories has been assessed using comparable methods within a new sample of patients with LBP, and the analysis shows that the previous findings of 4 trajectories^15^ of LBP have good external validity. For the first time, prognostic factors for trajectory membership have been described using data from a time point before the trajectories were derived. Findings indicate that socioeconomic status, pain intensity, and several dimensions of patients' illness perceptions (including consequences, emotional response, timeline, personal control, and passive behavioural coping) are key predictors of pain trajectory 5 years later.

A strength of this study is the prospective design, meaning that the measurement of prognostic factors associated with 5-year trajectory clusters clearly preceded the data collection period used to derive the trajectories. The use of pain trajectories as the outcome in the analysis of prognostic factors is also a strength because studies have shown that trajectories are more accurate measures of pain status than single or scattered follow-up points,^1^ and this type of analysis has been recommended.^25^ Our analyses using the single pain score at 5 years as the outcome generated fewer associations with the baseline prognostic factors. Trajectories of pain in this group of patients with back pain were relatively stable over time. However, this may not be the case in other groups of patients with pain, for example, patients with new episodes of back pain, pain in other body sites, or different age groups. For example, common trajectories of pain in knee osteoarthritis included both improvement and deterioration,^34^ as did pain across several sites in adolescents.^17^ These trajectories can only be captured by repeated measurements. Although repeated monthly pain assessments involve increased measurement burden for patients, it better reflects patterns of pain over time and reduces recall bias.^1^ New data collection methods such as web-based questionnaires, mobile devices, and the visual trajectories questionnaire for pain^12^ may be helpful to reduce the measurement burden. There were missing monthly pain scores within the sample used in our analysis; however, analysis of just those with no missing data did not affect the prevalence of each cluster and slightly increased the mean posterior probabilities for the assigned cluster. The long-term follow-up and use of validated questionnaires are also strengths. However, the sample size for the analysis of predictors was limited due to loss to follow-up at 5 years and the small size of some of the trajectories. Comparison with study participants not included in the full analyses or the whole cohort subjects^19^ showed few differences other than included participants were slightly older. Ideally, we would have kept the fluctuating cluster as a separate group when exploring cluster predictors; however, given the small number of participants in this cluster, this was not possible. Our study shows that the trajectories identified in another sample of back pain consulters appear generalizable but further work should assess the generalisability of the identified predictors for these trajectories, in particular whether a fluctuating pattern of pain has different predictors to a persistent severe pattern.

We allocated participants to the 4 trajectories of LBP derived in a previous study^15^ and assessed how well these participants fitted their allocated trajectory. An alternative approach to assess the generalizability of the previously derived trajectories would have been to derive the trajectories for this cohort using the same modelling method used in the previous study. However, deciding on the optimal number of clusters may have then been influenced by knowledge of these prior trajectories, given there is no definitive method using statistical goodness of fit measures of determining the optimal number of clusters.^25^ Hence, we performed this as a sensitivity analysis which again indicated good generalisability of the clusters. The approach we have taken utilises a strength of latent class analysis of using information on people with established and validated clusters to identify the most likely cluster membership of a new group of people. This approach has shown that a distinct group of patients with LBP could be clearly allocated to the same trajectories identified previously. Our study suggests that these trajectories can now be applied more widely in research for classifying back pain consulters.

Our findings on the predictors of cluster membership have similarities with other studies of associations with back pain trajectories. For example, Macedo et al^29^ reported that disability and self-efficacy were associated with trajectories, and Axén et al^2^ reported that pain intensity and duration were associated with trajectories, although in neither of these studies did the measurement of predictors clearly precede the derivation of trajectories. Other prognostic factors such as social class status and patients' perceptions about back pain have not been identified in previous trajectory studies. The latter finding supports the idea that people develop personal beliefs about their LBP and these influence subsequent reactions and behavior, which then may affect their long-term outcomes. Identification of these factors has potential clinical impact because these perceptions are modifiable factors and could be revised, for example, through education or cognitive restructuring.

The findings from this analysis that LBP trajectories have good external validity, combined with findings from previous studies showing the clearly different characteristics of patients in these trajectories,^15^ and their long-term persistence, have key implications. Knowledge of these long-term trajectories should enable better understanding of the long-term course of LBP. If the trajectory that an individual is likely to belong to can be identified, the challenge is then to identify effective management tailored to individual trajectories. This may mean more intensive treatment for those on a more severe trajectory, but for those likely to be in the milder trajectories, this may mean avoiding unnecessary investigations or overtreating. However, the finding that pain intensity at baseline predicts pain trajectory 5 years later, along with previous findings that trajectory membership^11^ and presence of LBP^28^ have long-term stability, indicates the challenge of shifting patients from more severe trajectories, and helping people better manage and cope with their symptoms may be the best current alternative. Improved understanding of how people get into these stable pain trajectories in the first place is required. Given the evidence of relatively trajectory stability in adult back pain populations, 1 potential direction would be a focus on children or young adult populations as a way of developing preventative interventions.^14^

Our results provide clear evidence of the generalizability of LBP trajectories in patients consulting in primary care and provide direction for future research and clinical practice.

## Conflict of interest statement

The authors have no conflicts of interest to declare.

This work was supported by the following grants: Arthritis Research UK [13413], the Wellcome Trust [083572], and the Medical Research Council Prognosis Research Strategy (PROGRESS) Partnership [G0902393/99558]. Time from NEF was supported by an NIHR Research Professorship (NIHR-RP-011-015). NEF is an NIHR Senior Investigator. The views expressed are those of the authors and not necessarily those of the NHS, the NIHR, or the Department of Health.

## Supplementary Material

SUPPLEMENTARY MATERIAL

## Appendix A. Supplemental digital content

Supplemental digital content associated with this article can be found online at http://links.lww.com/PAIN/A499.
